# Precise genotyping and recombination detection of Enterovirus

**DOI:** 10.1186/1471-2164-16-S12-S8

**Published:** 2015-12-09

**Authors:** Chieh-Hua Lin, Yu-Bin Wang, Shu-Hwa Chen, Chao Agnes Hsiung, Chung-Yen Lin

**Affiliations:** 1 Division of Biostatistics and Bioinformatics, Institute of Population Health Sciences, National Health Research Institutes, Zhunan, Miaoli County, 35053, Taiwan; 2 Institute of Bioinformatics and Structural Biology, National Tsing Hua University, Hsinchu, 30013, Taiwan; 3 Institute of Information Science, Academia Sinica, 128 Academia Road, Section 2, Nankang, Taipei, 11529, Taiwan; 4 Institute of Zoology, National Taiwan University, No. 1, Sec. 4, Roosevelt Rd., Taipei, 10617, Taiwan; 5 Institute of Fisheries Science, National Taiwan University, No. 1, Sec. 4, Roosevelt Rd., Taipei, 10617, Taiwan

## Abstract

Enteroviruses (EV) with different genotypes cause diverse infectious diseases in humans and mammals. A correct EV typing result is crucial for effective medical treatment and disease control; however, the emergence of novel viral strains has impaired the performance of available diagnostic tools. Here, we present a web-based tool, named EVIDENCE (**E**ntero**V**irus **I**n **DE**ep conception, http://symbiont.iis.sinica.edu.tw/evidence), for EV genotyping and recombination detection. We introduce the idea of using mixed-ranking scores to evaluate the fitness of prototypes based on relatedness and on the genome regions of interest. Using phylogenetic methods, the most possible genotype is determined based on the closest neighbor among the selected references. To detect possible recombination events, EVIDENCE calculates the sequence distance and phylogenetic relationship among sequences of all sliding windows scanning over the whole genome. Detected recombination events are plotted in an interactive figure for viewing of fine details. In addition, all EV sequences available in GenBank were collected and revised using the latest classification and nomenclature of EV in EVIDENCE. These sequences are built into the database and are retrieved in an indexed catalog, or can be searched for by keywords or by sequence similarity. EVIDENCE is the first web-based tool containing pipelines for genotyping and recombination detection, with updated, built-in, and complete reference sequences to improve sensitivity and specificity. The use of EVIDENCE can accelerate genotype identification, aiding clinical diagnosis and enhancing our understanding of EV evolution.

## Introduction

The *Enterovirus *(EV) genus (family ***Picornaviridae***) contains twelve species, including *Enterovirus A *to *H *and *J*, and *Rhinovirus A *to *C*. These viruses cause a wide range of diseases in humans and mammals. The single-stranded RNA genome of EV contains a single open reading frame (ORF) flanked by 5' and 3' untranslated regions (UTRs). The ORF encodes a polyprotein, which is further processed into 11 proteins: VP1-4 (structural proteins), and 2A-2C and 3A-3D (non-structural proteins) [1]. The genetic diversity of EVs arises from the accumulation of single-base changes during viral propagation, as well as from recombination events that cause genome segments to be swapped between or within EV genotypes. To date, 308 *Enterovirus *genotypes have been reported (http://www.picornaviridae.com/enterovirus/enterovirus.htm, on 2015/04), and the number is rising.

Different enterovirus genotypes cause different clinical symptoms [1]. Classical serotyping methods, such as serum neutralizing test and immunofluorescent assay, are not sufficient to specify all genotypes. For example, Tsao *et al*. (2010) reported that 15~30% of EV isolates failed to be serotyped in Taiwan [2]. To overcome this problem, many clinicians have turned to sequence-based molecular typing methods, which assign viral genotypes based on nucleotide sequences; such techniques are more successful at resolving EV isolates to the corresponding genotype, and also provide rapid diagnosis [3]. The *VP1 *capsid-coding region has been suggested to be the most suitable region for EV genome genotyping [4,5]. In addition, the 5'UTR [6,7], *VP2 *[8,9], *VP4 *[10,11] and *3D *[11,12] regions, as well as combinations of more than two regions, including the 5'UTR and *VP4/VP2 *[13], the 5'UTR and *VP1*[14], and *VP1 *and *3D *[15], have been evaluated for their usefulness for improving the sensitivity and specificity of diagnosis. However, incongruent results may be obtained from different typing methods based on either single or multiple coding regions of the genome [16-19].

At present, there are two EV genotyping tools: enterovirus genotyping tool (version 0.1; National Institute of Public Health and the Environment (RIVM), the Netherlands) [20] and the genotyping tool of the NCBI [21]. Both of these resolve genotypes on the *VP1 *region, and disregard the rest of the EV genome. This approach limits the ability to distinguish between strains that originated from recombination events. Moreover, EV genotype reference sequences are never updated in these libraries.

A fast, highly sensitive, and specific molecular typing tool is essential for clinical diagnosis and medical treatment. In this study, we developed a web tool, EVIDENCE (EnteroVirus In DEep coNCEption), a workbench for phylogenetic-based genotyping and recombination detection in EV genomes. Up-to-date EV classification data, nomenclature, and GenBank accession numbers for each genotype's prototype sequence were collected from the Picornaviridae Study Group website at http://www.picornaviridae.com/[ 22], and these were combined with sequences collected from the NCBI to build the genotyping reference set (GTRefSet). Phylogenetic inference was used to resolve the best-fit genotype of novel EV sequences using single or multiple genomic regions of interest. For detection of recombination events, the closeness between the suspected recombinant and reference sequences was measured as bootscanning supports by the phylogenetic method, and as sequence similarity by the distance method. The pipeline design enables users to seamlessly run recombination analyses with guidance for the choice of references. Furthermore, we revised the EV sequences in GenBank to standardize the nomenclature and to clarify genotype assignments. The collected sequences were built into the database, and can be retrieved in an indexed catalog or be searched for by keyword or sequence similarity.

EVIDENCE is the first web-based tool providing pipelines for genotyping and recombination detection based on both sequence context and phylogenetic inference. Furthermore, EVIDENCE uses the most complete and regularly updated reference sequences to maintain high sensitivity and specificity, thereby accelerating genotype identification in clinical diagnosis and enhancing our understanding of EV evolution. EVIDENCE is available at http://symbiont.iis.sinica.edu.tw/evidence.

## Materials and methods

### Reference sequence sets

Nucleotide sequences of EV prototype strains (Additional file 1: Table S1) listed in the Picornaviridae Study Group website (http://www.picornaviridae.com/) were fetched from GenBank database. If the complete genome of a prototype strain was not available, we collected sequences of all the other available regions instead. For a genotype without a reference prototype assignment, the longest and/or earliest reported sequence was selected as the genotype's representative reference. Finally, 396 nucleotide sequences are selected to build the three hundred and eight prototype models (the genotyping reference set, GTRefSet). Furthermore, we expanded the prototype GTRefSet to the extended reference set (ExRefSet, Additional file 1: Table S2) by including the sequences that is highly similar (sequences identity >75%) [23] to the representative prototype sequence of the same genotype. GTRefSet was the core reference set for phylogenetic analysis and recombination analysis, and ExRefSet was used for automatic re-assignment of EV sequence genotypes.

### Re-classification of all EV sequences in GenBank

**Figure 1:**
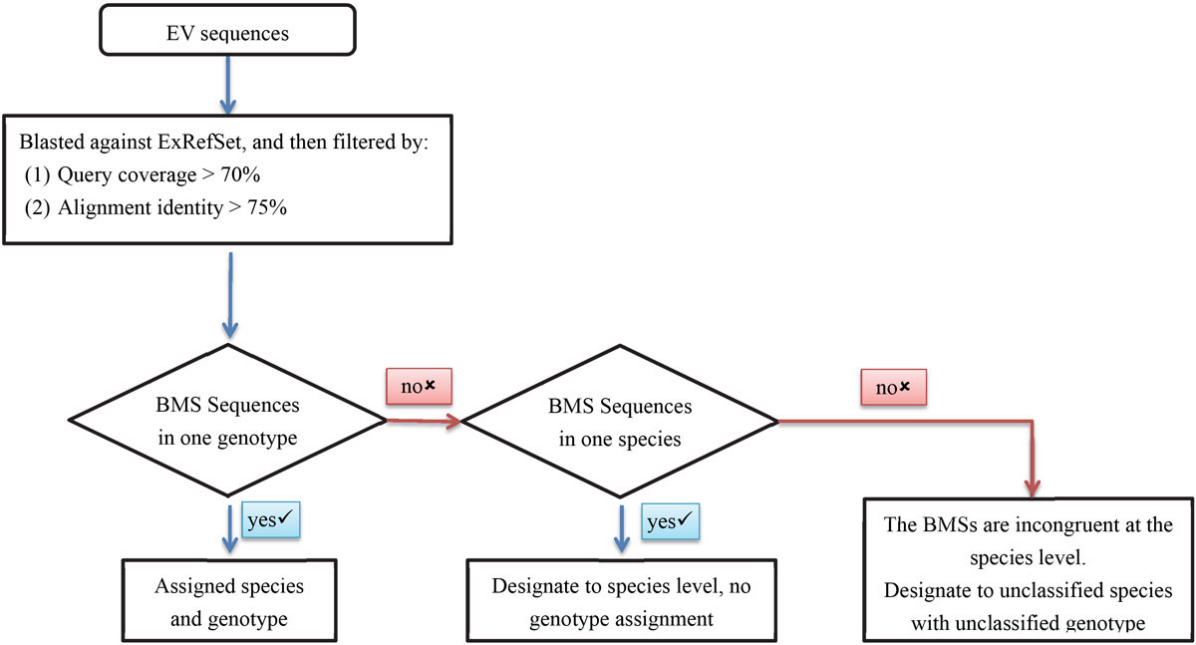
**Genotype re-classification procedures**.

Nucleotide sequences from the *Enterovirus *genus deposited in GenBank (release 206) were collected, with the exception of sequences that are denoted as being from environmental samples. To unify the sequence taxonomical nomenclature in accordance with the decisions of the International Committee on Virus Taxonomy (ICTV) 2014 [24,25] and to identify sequences with potentially misassigned genotypes, the collected EV sequences were run through the following procedures (Figure 1). First, we performed a BLAST search (dc-megablast, BLAST+ package, version 2.2.28) [26] using a query sequence *q *against reference sequence *x *in ExRefSet (*E*), and extracted the following information: BLAST raw score (*braw_qx_*), % of query coverage per reference sequence (*cov_qx_*), and % identity (*identity_qx_*). *Cq *is a subset of *E *with all high blast-scored alignments to *q*,

$Cq=\left\{x\in E\left|\;cov_{qx}>70\%,\;identity_{qx}>75\%\right)\right\}$.

Let *rank*(*braw_qx_*),*rank*(*cov_qx_*),*rank*(*identity_qx_*) be the ascending sorting rank of BLAST raw score, alignment coverage, and identity between *q *and *x *in *Cq*, respectively. We build a set of the rank sums *rS_Cq _*where

$rs_{Cq}=\left\{rank\left(braw_{qx}\right)+rank\left(cov_{qx}\right)+rank\left(identity_{qx}\right)\left|x\in Cq\right)\right\}$.

Finally, the best mixed-ranking score (**BMS**) for the query *q *is defined as the largest rank sum *r *in *rS_Cq_*,

$BMS_{q}=\text{max}\left(r\left|r\in \right)rs_{cq}\right)$.

Sequences are assigned to the genotype/species of their BMS references if the BMS is only reached by one reference sequence or multiple references with consistent genotype/prototype assignment. If the BMS of a query sequence is reached by two or more different genotypes in one species, the nomenclature assignment of this sequence is set to the species level. Query sequences are assigned as "unclassified" species with "unclassified" genotype if a BMS from a highly related sequence is not detected in ExRefSet (e.g., ***Cq ***is an empty set), or multiple references reach the BMS but are incongruent at the species level.

The rationales of using three parameters in the mixed-ranking score function are described below. The expectation value (E-value) given by BLAST is often used to indicate the significance of an alignment, and is often inferred to the homology/similarity relationship of the hit to the query sequences. Calculation of E-value is affected by the content of searching database and the length of the matching segment. For example, a small E-value may be granted to a short region in high sequence similarity, leading to false positive results if we use an E-value cut-off for selecting sequence matches. In contrast, the BLAST raw score is directly derived from alignment segments can eliminate this artifact [27]. Thus, we use BLAST raw score instead. The BLAST raw score is also depended on the scoring parameters being used (*i.e.*, reward for matching base and penalty for mismatching base/gap) [28,29]. In our case, the scoring parameters are using the defaults in discontiguous megablast (dc-megablast), says, match = 2, mismatch = −3, gap open = −5, and gap extension = −2. This empirical setting is optimized for catching highly similar sequences with gap allowance. Then, we calculated the BLAST raw score for the query sequence *q *to the reference *x *in the reference set, and granted the rank score *Braw_qx _*to each *q-x *pair by the sorting order of the score.

Furthermore, we adopted the percentage of identity and the coverage of the alignment in the algorithm to address the importance of the conservation of the base components and the overall alignments of any two sequences in comparison. These two indexes are necessary for identifying the closest homologous sequences. The numeric values of the identity and coverage were transformed to ranked scores, in which we can sum up for the importance evaluated by three different scoring scheme in a normalized scale. Therefore, the mixed-ranking score is based on a measure of sequence similarity, adding weights on the importance of the quality of alignment.

### Phylogenetic-based genotyping procedure

**Figure 2:**
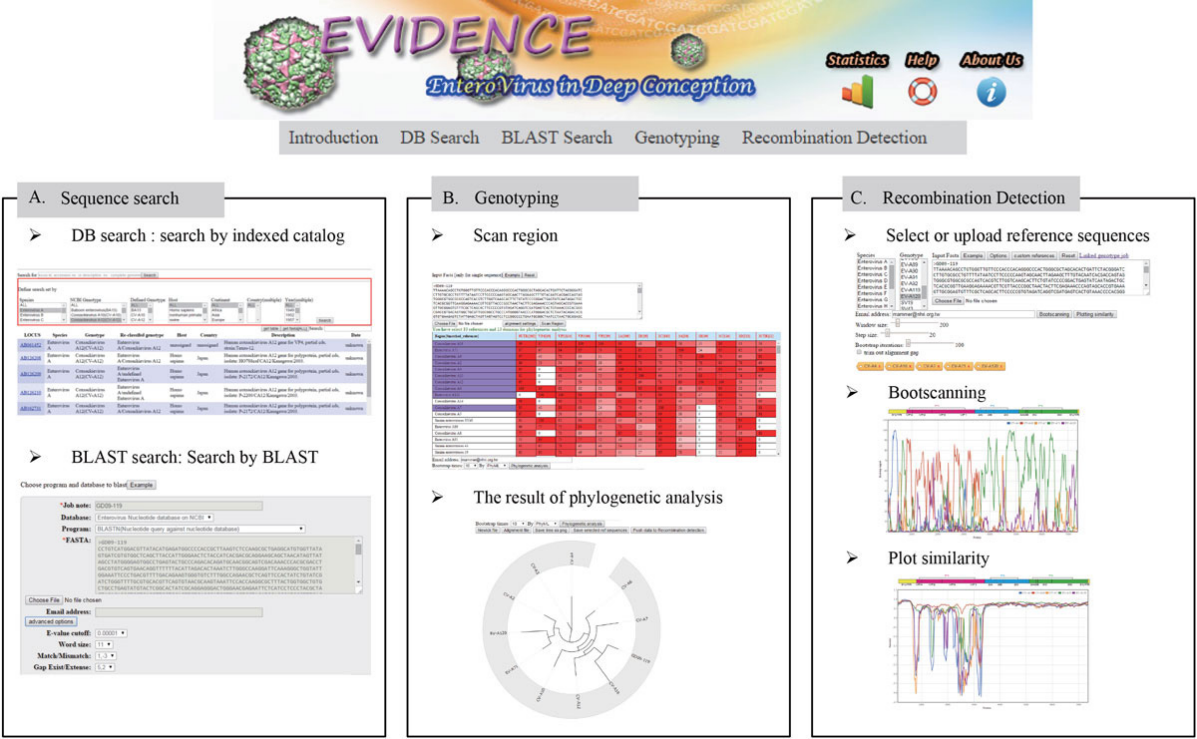
**EVIDENCE interface**. (A) **Sequence Search**. In DB search, keywords or multiple conditions can be used to search for related sequences. In BLAST search, single or multiple sequences are used to BLAST against all EV sequences to search for similar sequences. (B) **Genotyping**. First, the query sequence is compared to the GTRefSet (the scan region step). The result table contains indices of mixed-ranking scores presenting segment similarity. References for phylogenetic analysis are selected from this table. (C) **Recombination Detection**. The potential recombination events can be detected through observing swaps of the most similar reference (plot similarity) or changes of the relatively closest neighbor (bootscanning) in the genome segments.

EV prototypes in GTRefSet were further segmented into the thirteen genomic regions (5'UTR, 11 segments corresponding to the mature peptide-coding regions, and 3'UTR) according to the coordinates described in their original GenBank documents. For sequences without this information, we used NetPicoRNA [30] to predict the polyprotein cleavage sites and then manually curated ambiguous boundaries. The phylogenetic-based genotyping procedure consists of two steps: ***scan region ***and ***phylogenetic analysis***. In the ***scan region ***step, an input query sequence is compared to the GTRefSet members using BLAST. The index of the mixed-ranking score of each genome region of each reference to the query is then calculated (Figure 2). The region scan result table is sorted by the sum of mixed-ranking score ***r ***of all available genome regions; the reference sorting order is changed dynamically according to the genome regions selected.

Executing the ***phylogenetic analysis ***requires one query sequence and at least two reference strain prototype sequences. The genome regions of interest are cropped from the query sequence and concatenated for phylogenetic analysis. Multiple sequence alignments of the concatenated query and reference fragments are performed by Clustal Omega version 1.2.0 [31]. The phylogenetic inference was done in by PhyML 3.0 [32] (model GTR + G + I) with user defined bootstrap iterations. The circular phylogenetic tree topology is generated by jsPhyloSVG library [33].

### Recombination event detection

We implemented Bootscan [34] and SimPlot [35] methods in EVIDENCE to detect possible recombination events and graphically present the results. Briefly, multiple sequence alignments between the query and reference genotype sequences (built into GTRefSet or custom uploaded) were performed using Clustal Omeg. The distance and similarity between sequences are calculated using DNADIST in PHYLIP (package version 3.5c) [36] and phylogenetic inference is analyzed using the Neighbor-joining (NJ) method with the Kimura two-parameters substitution model. Parameters, including sliding window size, step size, bootstrapping iterations, and the use of trimA1 (version 1.2) [37] to remove gaps, are adjustable. For each sliding window, the bootstrap value of the reference that was the first neighbor clustered to the query was used to derive the percentage of bootstrap support. Finally, profiles of query-reference closeness, evaluated by similarity or percentage of bootstrap support of each query-reference pair in each sliding window segment, were plotted along the EV genome. A clear crossing-over of two query-reference profiles with sharp slopes suggests a swap of best-fit reference and the presence of a nearby recombination breaking point.

### System framework

EVIDENCE (http://symbiont.iis.sinica.edu.tw/evidence/) is constructed on an open-source Linux Arch (version), Nginx (version 0.12.4), and SQLAlchemy and SQLite relational database (version 3.8.4.3) structure. Graphical visualization was provided using Canvas and SVG library. Scripts for joining software packages to seamless pipelines were written in Perl and Python. The whole system is run in a virtual machine (CPUs of 2.27GHz, 8 cores, 16 GB RAM) located in the Institute of Information Science, Academia Sinica, Taiwan.

## Results and discussion

### The usage of EVIDENCE

EVIDENCE is a searchable database for updated and re-classified EV sequences and a workbench for EV genotyping and recombination detection (Figure 2).

#### Database search

A total of 54,790 up-to-date EV sequences were curated and built into an indexed category of virus species, genotype, and epidemiological annotations (host, continent, country, and the reported year). This database can be browsed through the hierarchical structure or searched by keywords (Figure 2A, panel 1) or sequence similarity (e.g., BLASTN, TBLASTN, or TBLASTX) (Figure 2A, panel 2). For both sequence search functions, the basic features extracted from GenBank records and reclassified genotypes are shown in table format. The search results, including the brief table of reported entries and the FASTA file of hits, are made available for download.

#### Genotyping

EVIDENCE matches query sequences to references in GTRefSet using the BLAST algorithm (Figure 2B, panel 1). To perform genotyping, the query nucleotide sequence in FASTA format is pasted or uploaded through the sequence input interface, and then submitted by clicking on 'scan region'. Scores for the query to each EV genomic region of each prototype are calculated and displayed in the results table. The table functions like a flexible input interface for the next phylogenetic analysis step. Clicking on the table column (the genome region) or table row (sequence title) will select the region or the references, respectively. The table is decreasingly sorted by the mixed-ranking score of a single column, or by the sum of mixed-ranking scores of all the selected columns (Figure 2B). The sorting order implies the relative fitness of each query-reference pair with respect to the region(s) of interest.

The phylogenetic analysis step calculates the relatedness between the query and the selected references. A tree topology is generated by PhyML with adjustable bootstrap iterations (default: 100 iterations). Phylogenetic analysis outputs, including the tree topology as a newick file and a png file, multiple sequence alignment, and the selected reference sequences, are made available for download (Figure 2B).

#### Recombination

The basic principle for detecting potential recombination events is to segment the whole EV genome into small overlapping segments, in order to identify swaps of the most similar reference (plot similarity) or changes in the relatively closest neighbor (bootscanning) in the successive segments; similarity is derived from the sequence distance for each query-reference pair, and sequence neighbors are determined based on the percentage of bootstrap iterations supporting the reference as the closest neighbor to the query.

The recombination analysis takes a single sequence query, accompanied with three or more references to give adequate estimations. The reference strains are selected from those built into GTRefSet and/or from uploaded 'custom references'. The parameters for phylogenetic inference include sliding window (default: 200), step size (default: 20), bootstrapping (default: 100 iterations), and whether or not gaps in the multiple sequence alignment are trimmed (default: no trimming); all of these parameters are adjustable (Figure 2C, panel 1).

Plotting of the similarity or bootstrapping results is optional. The EV genome diagram is plotted on top to help visualize the location of recombination events. Dynamic figures are used to present the data and allow the user to zoom into/out of the plot. The value of each sliding window on the plot is shown via mouseover events, and the user may zoom in on a specific region of the plot by cropping the region through mouse dragging (Figure 2C, panel 2). The bootscanning/similarity plot, multiple alignment table, and bootstrap/similarity value table can be downloaded.

The choice of reference strains for recombination detection is crucial. Using inappropriate reference strains to identify the recombination region may eliminate the significance of the bootscanning result, and increase the noise of recombination breakpoint determination. In EVIDENCE, users can start the analysis from genotyping. References in GTRefSet are evaluated for the fitness of each genome region, which can help users select appropriate regions. The data, including the query sequence and at least three selected references, are redirected by clicking "push data to recombination detection" after the phylogenetic analysis step.

It is worth noting that using too many reference sequences in a recombination detection analysis may return an insignificant bootscanning plot or a messy similarity plot. We suggest that analysis should begin with less than ten references, and then the non-informative ones should be removed to improve the resolution.

### Statistics of GTRefSet and ExtRefSet

**Figure 3:**
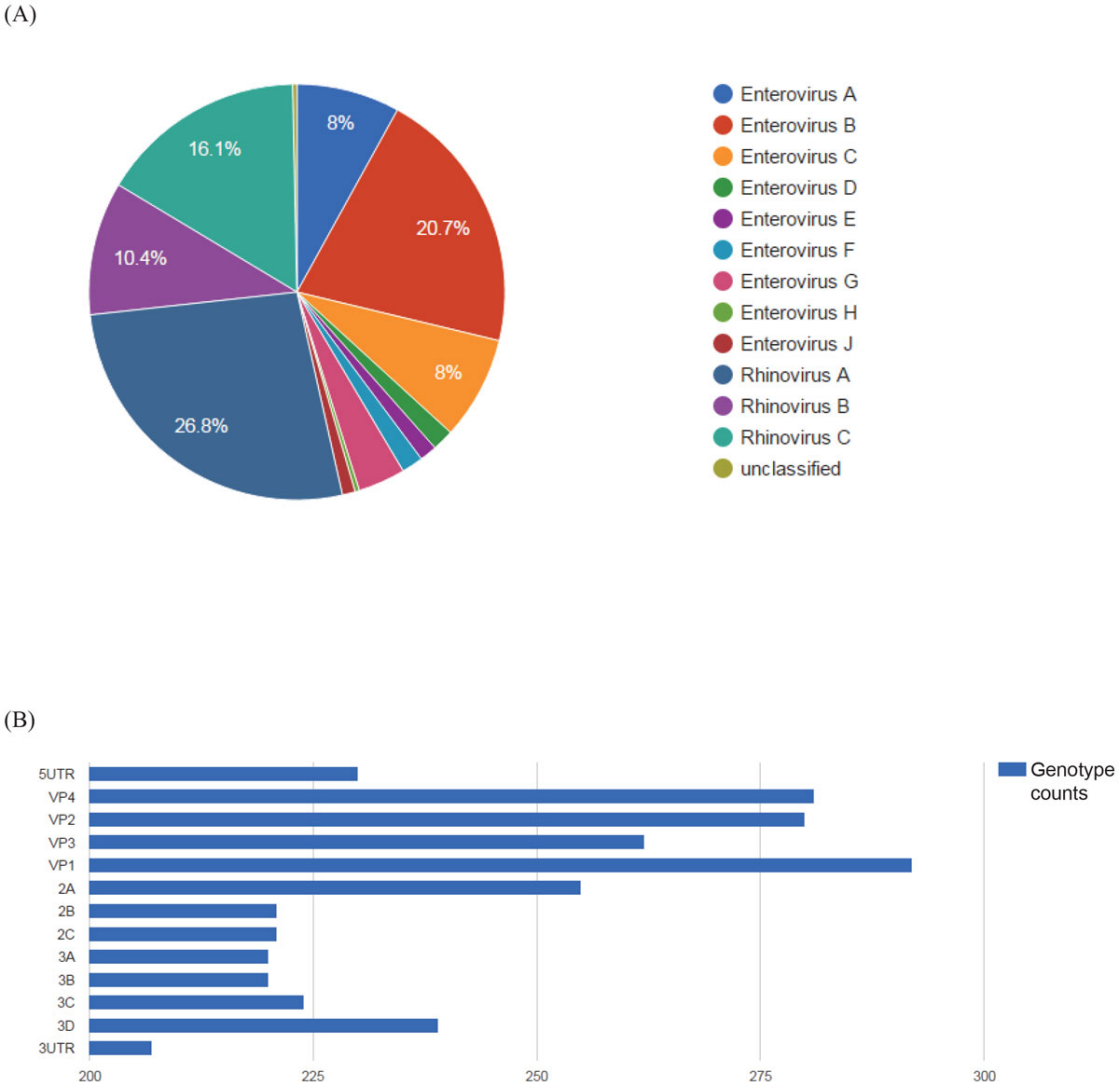
**Statistics of GTRefSet**. (A) The species composition of the database. Enterovirus B and Rhinovirus A collectively contribute to about half of the total known genotypes. (B) Genome region view.

The genotyping reference set (GTRefSet) of 308 reported prototype models includes 204 full genomes, 17 partial genomes, and 69 partial segments (data in 2015/04). Eighteen genotypes are not related to any reference sequence in the database and will not be included in the present EVIDENCE reference sets. Figure 3 show statistics for GTRefSet, by species and by genome region. Of the 13 genome regions, VP1 is the most sequenced; the VP1 regions of almost every prototype were reported. By selecting sequences with an identity >75% to the representative prototype sequence of the same genotype, 143 full genomes, 26 partial genomes, and one fragment were appended to form ExtRefSet (Supplementary Table S2, See Additional file 1).

### Updating the classification of sequences using a BLAST-based method

To date, 54,790 EV sequences are deposited in GenBank, but the genotype assignment of these sequences in GenBank may be inaccurate. One possible reason for any inaccuracies is that the genotype of a sequence is assigned by individual researchers upon submission. However, differences in the typing methods used may lead to different conclusions. As mentioned above, serological assays and molecular typing may generate conflicting results if the examined strain has undergone a recombination event that changed the viral genome and the correlation of epitopes with the reference virus strain; such inconsistency is independent of the specificity of the antibody used to resolve strains at the genotype level. A second possible reason is that the nomenclature and the virus classification system are not synchronized between reference databases. Virus classification for NCBI taxonomy is based on that of the International Nucleotide Sequence Database Collaboration (INSDC) [38]. However, classification of viruses is less stable than that of other organism kingdoms due to the rapidly evolving nature of these infectious life forms. Irregular updates have led to the inconsistent nomenclature and taxonomic classification used by GenBank and the International Committee on Virus Taxonomy (ICTV). Moreover, the updated viral classification scheme has not been applied to sequences previously deposited in the database.

In order to unify the nomenclature and taxonomic scheme of EV in accordance with the release by the ICTV (2014), we revised the classification of EV sequences collected from GenBank. To include the intragenic genetic variation of a genotype, we used ExRefSet instead of GTRefSet to enhance the sensitivity of the BLAST-based method. The re-classification results showed high congruence with GenBank classification (Table 1; details on the congruence of each genotype are provided in Additional file 1: Table S3). For example, sequences in the well-studied and largest genotype, EV-A71, comprised 16.12% of the EV-related sequences in GenBank, and over 99% sequences in this genotype remained in their original assignment after re-classification. In addition, 633 EV sequences were newly assigned to EV-A71. About 1/3 of these re-classified sequences were isolated from the EV-A71 strain or identified as EV-A71 [39-49], but were assigned to the species level (Enterovirus A) or misassigned to other genotypes; 68.8% of the remaining sequences (308 sequences) were typed using the amplicons derived from universal 5'UTR primer for all enteroviruses (Table 2) [14,50-55]. It have been reported that the 5' UTR regions of *Enterovirus A *and *Enterovirus B *are indistinguishable due to their highly conserved secondary structures [7,56,57]. Therefore, the use of certain genotyping techniques may generate inadequate genotype assignments, resulting in the inconsistencies in assignment observed between those of the original records and those obtained with EVIDENCE.

**Table 1:** 

Species	GenBank Nomenclature	Assigned by EVIDENCE	Sequences with Congruent Assignment (% *)
Enterovirus A	15875	16057	15794 (99.49%)
Enterovirus B	16432	16502	16238 (98.82%)
Enterovirus C	8224	8202	8146 (99.05%)
Enterovirus D	1031	1132	1031 (100%)
Enterovirus E	73	43	41 (56.16%)
Enterovirus F	5	43	5 (100%)
Enterovirus G	196	216	190 (96.94%)
Enterovirus H	10	10	10 (100%)
Enterovirus J	9	11	5 (55.56%)
Rhinovirus A	2733	5088	2697 (98.68%)
Rhinovirus B	608	1101	592 (97.37%)
Rhinovirus C	2309	3690	2259 (97.83%)
unclassified Enterovirus unclassified Rhinovirus	4957	367	119 (7.4%)

Remark: * The percentage is calculated as follows:$\frac{\text{Sequences}\;\text{with}\;\text{Congruent}\;\text{Assignment}}{\text{Sequences}\;\text{in}\;\text{GenBank}\;\text{nomenclature}}\times 100\%$

**Table 2:** 

Original Species	Original Genotype	#Sequences	Region^2 ^(sequence number)
Enterovirus A	Coxsackievirus A2	11	5'UTR (1), 2C (2), 3D (8)
Enterovirus A	Coxsackievirus A3	2	5'UTR (1), 3D (1)
Enterovirus A	Coxsackievirus A4	13	2C (2), 3D (11)
Enterovirus A	Coxsackievirus A5	2	3D (2)
Enterovirus A	Coxsackievirus A6	74	5'UTR (69), 2C (2), 3D (3)
Enterovirus A	Coxsackievirus A7	7	2C (2), 3D (5)
Enterovirus A	Coxsackievirus A8	6	2C (1), 3D (5)
Enterovirus A	Coxsackievirus A10	94	5'UTR (85), VP4-VP2 (2), 2C (3), 3D (4)
Enterovirus A	Coxsackievirus A14	3	VP1 (1), 2C (1), 3D (1)
Enterovirus A	Coxsackievirus A16	1	3D (1)
Enterovirus A	Enterovirus A76	1	3D (1)
Enterovirus A	NA^1^	54	5'UTR (28), 2BC (2), 3D (24)
Enterovirus B	Echovirus 4	3	5'UTR (3)
Enterovirus B	Echovirus 9	5	5'UTR (5)
Enterovirus B	NA^1^	10	5'UTR (10)
unclassified Enterovirus	UE^1^	22	5'UTR (10), VP4 (3), VP1 (3), 3D (6)

Remarks:

1. Abbreviations in use: NA, Not assigned; UE, including all classes under unclassified Enterovirus.

2. The sequenced region is a partial or complete segment.

We further compared the performance of our reclassification procedure with that of the RIVM EV genotyping tool (http://www.rivm.nl/mpf/enterovirus/typingtool), a phylogenetic-based typing tool which uses a partial VP1 region and the neighbor-joining (NJ) method with HKY85 or TamNei model, using the sequences identified and typed by traditional neutralization methods [58-61] as a gold standard. As shown in Table 3 EVIDENCE generated more consistent results with antigenic typing. For example, one untyped human rhinovirus (HRV) partial VP1 sequence (GenBank accession number AF152281) has 89.37% similarity to HRV-A31; RIVM genotyping tools assigned it to the HRV species, whereas EVIDENCE returned HRV-A31 as the genetically closest genotype.

**Table 3:** 

Species	#Sequence	Region	#Genotype	Number (%) of Genotype Discrepancies		References
					
				EVIDENCE	RIVM Genotyping Tool	
**Enterovirus A**	49	5'UTR	2	0	49 (100%) (49 only typed to species level)	[72]
	59	VP1	8	0	0	[27,28,52,53]
	3	CG	3	0	0	[53]
**Enterovirus B**	101	VP1/VP2	22	6 (5.9%) (4 seqs typed to species level, 2 mistyped)	6 (5.9%) (4 seqs typed to species level, 2 mistyped)	[27,28,52]
**Enterovirus C**	13	VP1	6	1 (7.7%) (mistyped)	2 (15.4%) (1 seq typed to species level, 1 mistyped)	[27,28]
**Rhinovirus A**	1	VP1	1	0	1 (100%) (1 seq typed to species level)	[28]
**Untypeable**	5	VP1	NA	5 (100%) (5 successfully typed)	5 (100%) (1 seq typed to species level, 4 successfully typed)	[28]

### A case study

Here, we demonstrate a genotyping and recombination pipeline using two coxsackievirus A16 (CV-A16) strains with distinct pathogenesis.

Coxsackievirus strains CV-A16 GD09/24 (GenBank accession KC117317) and GD09/119 (GenBank accession KC117318), exhibiting differing levels of clinical virulence, were isolated in Guangdong, China, in 2009 [62]. The authors performed phylogenetic analysis with 28 CV-A16 homologous strains and one EV-A71 prototype strain to assign GD09/24 and GD09/119 to the CV-A16 genotype. To detect recombination events, the authors compared two novel CA-A16 strains with two EV-A71 strains and one CV-A16 prototype strain. Bootscanning results indicated that GD09/24 and GD09/119 underwent homologous recombination with EV-A71 in the P2 and P3 regions.

**Figure 4:**
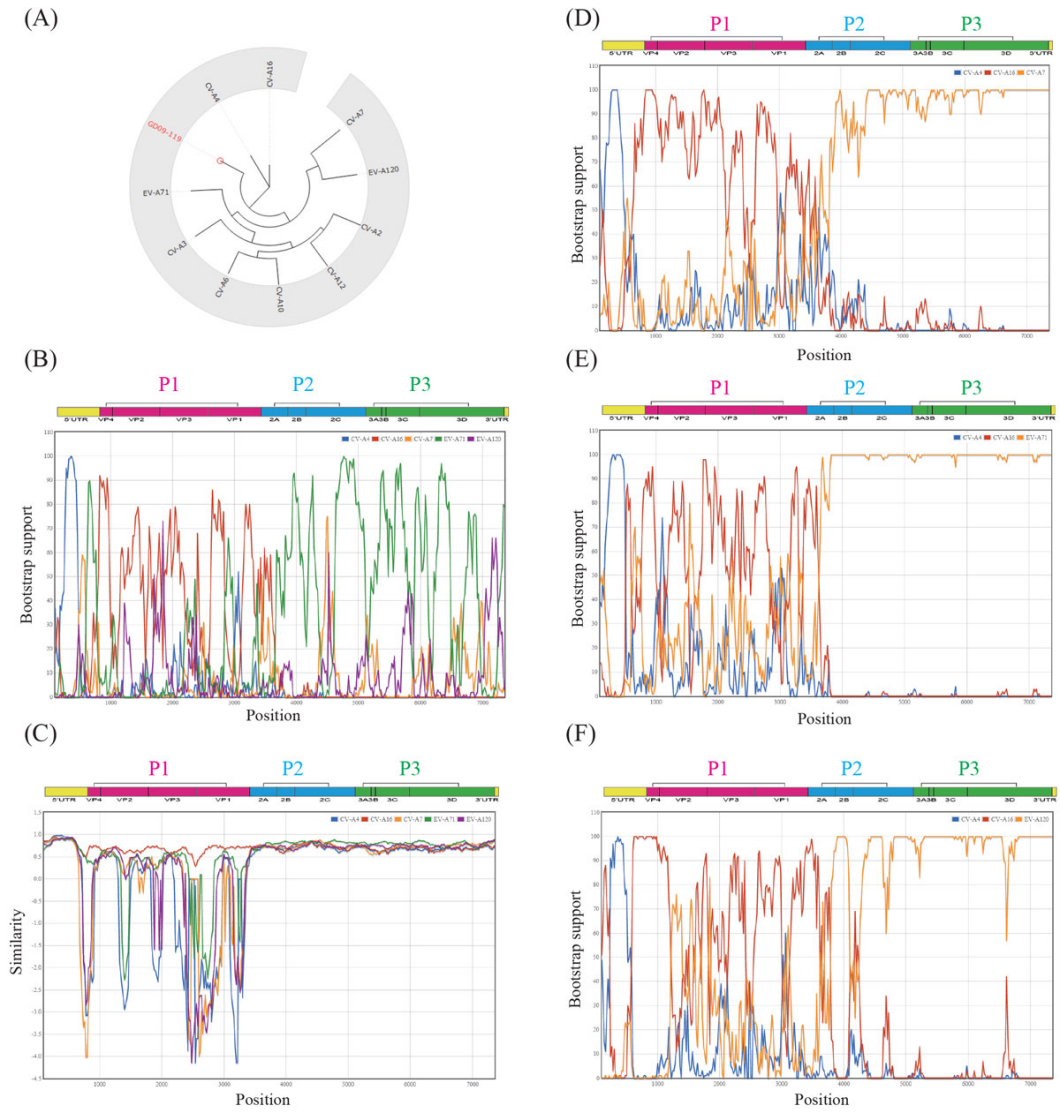
**Detection of recombination events in a highly virulent enterovirus strain CA16/GD09/119**. The ML tree of the GD09/119 complete genome sequence to the ten top ranked prototype references in GTRefSet is shown in panel A. The five closest references (CV-A4, CV-A16, CV-A7, EV-A71, and EV-A120) were selected for recombination detection, which was plotted using Bootscanning (panel B) and similarity (panel C). The results of the potential recombination analyses of GD09/119 to CVA4, CVA16, and one of CV-A7 (panel D), EV-A71 (panel E), or EV-A120 (panel F), are shown as bootscanning plots.

In this study, we used our EVIDENCE analysis pipeline to study GD09/24 and GD09/119. First, the sequences were submitted for analysis by the genotyping tool. After the scan region step (alignment parameters at the default settings), reported references were sorted by the sum of mixed-ranking scores of all 13 genome regions. We selected all genome regions and the top 10 ranked references to perform phylogenetic analysis with 500 bootstraps. The phylogenetic tree topology indicated that the closest genotype to GD09/119 is CV-A16 (Figure 4A; Additional file 2: Figure S1 for GD09/24), which is consistent with the findings of the previous study. The five selected reference strains and the query were piped to the recombination detection page by clicking on the "push data to recombination detection" button. The bootscanning (Figure 4B) and similarity (Figure 4C) results revealed that GD09/119 is closely related to CV-A4 in the 5'UTR region, to CV-A16 in the P1 region, and to EV-A71, CV-A6, and CV-A7 in the P2 and P3 regions. GD09/24 showed the same recombination pattern (Additional file 2: Figure S1). In the earlier study [50], the authors used two references, EV-A71 and CV-A16, to detect recombination events. Thus, they did not observe the high correlation of 5'UTR to CV-A4 described here. In addition, it is difficult to determine the origin of the P2 and P3 regions of GD09/119 and GD09/24. EV-A71, CV-A6, and CV-A7 all showed high bootstrap values when individually subjected to bootstrapping with CV-A4 and CV-A16, supporting the hypothesis that recombination occurred in the P2 and P3 regions (Supplementary Figure S1, See Additional file 2). In fact, the P2 and P3 regions are highly conserved in EV-A71, CV-A6, and CV-A7. The results emphasize the limitations of phylogenetic-based recombination-detecting methods. The non-structural regions play major roles in viral replication, protein processing, virulence, and virus shedding [63-66], and thus influence host immune responses [67]. This may be the reason for the distinct pathogenicity of these two isolates. In addition, it has been reported that CV-A16 co-circulated and/or co-infected with EV-A71, CV-A6, or CV-A4 [68-71] in China from 2008 to 2014. Therefore, the novel epidemic strains of EV isolates GD09/24 and GD09/119 may have originated from recombination of CV-A16 to EV-A71, CV-A4, CV-A6, CV-A7, or EV-A120.

This demonstration shows that the genotyping and recombination pipeline in EVIDENCE can provide suitable candidates as references for recombination detection. Additionally, users can download all output files, and perform analyses using different reference sequences or genomic region(s) with a user-friendly interface.

## Conclusions

Classical EV typing is largely dependent on serotyping methods. VP1 has been the subject of extensive research on account of the neutralization potency of its antiserum [23,58,72]. Typing specificity can be improved by using a panel of antibodies against VP1 and other viral proteins [73-77]. Thus, genotypes may be assigned through observing the response of several antibodies raised from epitopes in different genome regions. If a novel EV strain emerged from a recombinant event that joined epitopes of different parent strains, the serological phenotype may fail to reflect clinical virulence. As more EV sequences are reported, it is increasingly apparent that recombination occurs frequently within inter- or intra- genotypes. Moreover, each genomic region is subject to distinct selective pressures, and thus their evolution is independent of one another [78,79]. Increased genetic diversity often leads to phenotypic variation, which is problematic for clinical therapy.

EVIDENCE can be used to perform EV typing based on sequence context. This tool disassociates reference prototypes into functional components of the virus genome, and performs analysis in a modularized manner. However, the correlation of individual genome regions with genome virulence remains unclear. We hope that EVIDENCE can be used to address this question and provide insights into EV evolution, as well as facilitate the diagnosis of clinical specimens to ensure appropriate treatment.

## Competing interests

The authors declare that they have no competing interests.

## Authors' contributions

CHL and CYL designed the algorithm, conducted the experiments, and drafted the manuscript together with SHC. CHL and YBW worked on EVIDENCE website construction and implemented the tool and phylogenetic analysis workflow. CYL, CAH and SHC participated in discussions and conceptualization as well as revising the draft. All the authors read and approved the manuscript.

## Supplementary Material

Additional file 1 Supplementary data files: Table S1: GTRefSet list, Table S2: ExRefSet list and Table S3: Summary of congruence between GenBank and re-classification in this study. Click here for file

Additional file 2 **Figure S1**. Detection of recombination events in a mild virulent enterovirus strain CA16/GD09/24. (*.pdf) Click here for file

## Declaration

The publication costs for this article were funded by Ministry of Science and Technology (MOST), Taiwan.

This article has been published as part of *BMC Genomics *Volume 16 Supplement 12, 2015: Joint 26th Genome Informatics Workshop and 14th International Conference on Bioinformatics: Genomics. The full contents of the supplement are available online at http://www.biomedcentral.com/bmcgenomics/supplements/16/S12.
