# Supplementary material for: Precise genotyping and recombination detection of Enterovirus
Source: BMC Genomics. 2015 Dec 9;16(Suppl 12):S8. doi: 10.1186/1471-2164-16-S12-S8 (PMC4682392; doi:10.1186/1471-2164-16-S12-S8)
Supplement: Additional file 2 — Figure S1. Detection of recombination events in a mild virulent enterovirus strain CA16/GD09/24. (*.pdf) [file 1471-2164-16-S12-S8-S2.pdf]

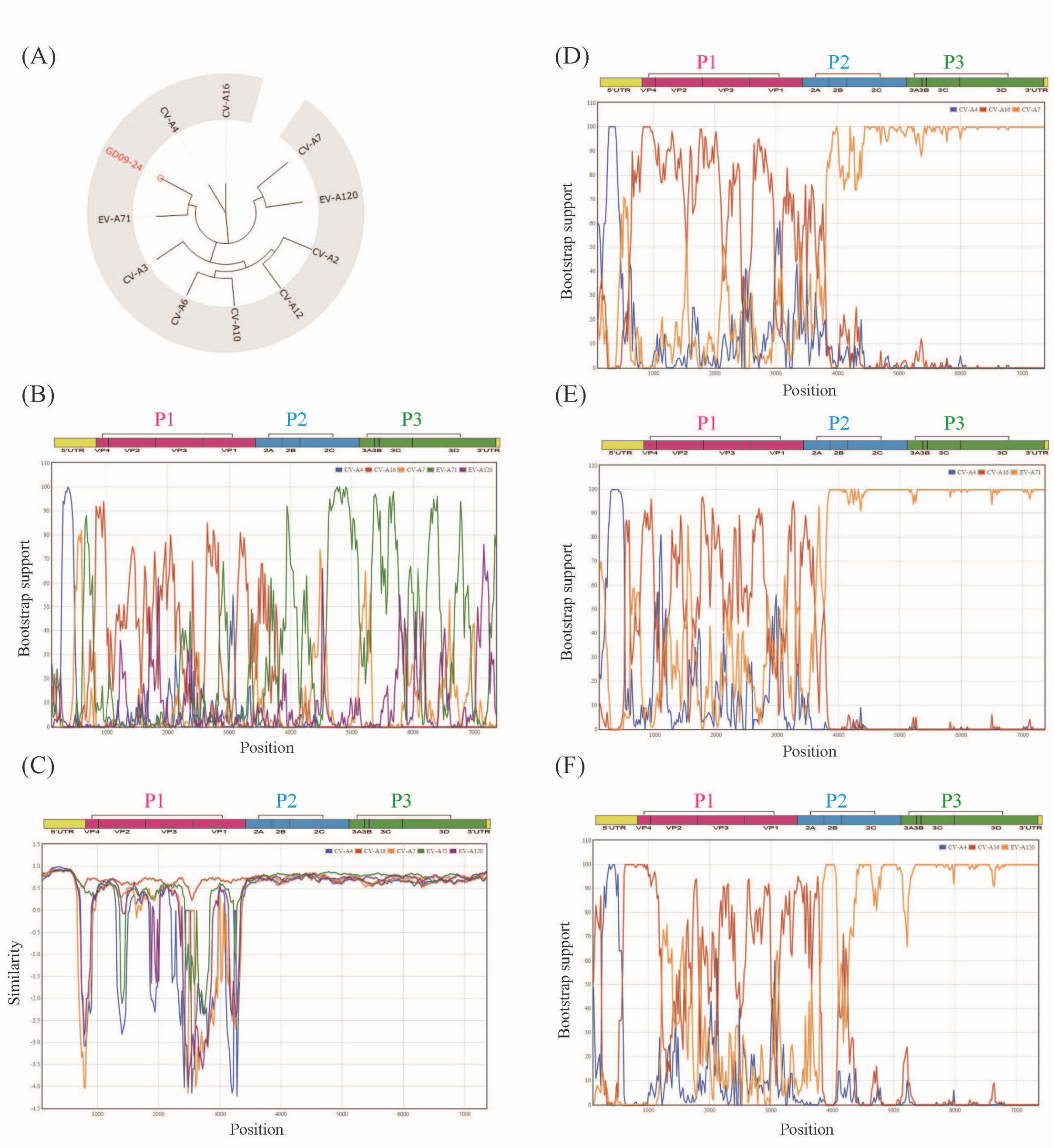

Figure S1. Detection of recombination events in a mild virulent enterovirus strain CA16/GD09/24. The ML tree of the GD09/24 complete genome sequence to the ten top ranked prototype references in GTRefSet is shown in panel A. The five closest references (CV-A4, CV-A16, CV-A7, EV-A71, and EV-A120) were selected for recombination detection, which was plotted using Bootscanning (panel B) and similarity (panel C). The results of the potential recombination analyses of GD09/119 to CVA4, CVA16, and one of CV-A7 (panel D), EV-A71 (panel E), or EV-A120 (panel F), are shown as bootscanning plots.
